# Belantamab Mafodotin in Patients with Relapsed/Refractory Multiple Myeloma: Results of the Compassionate Use or the Expanded Access Program in Spain

**DOI:** 10.3390/cancers15112964

**Published:** 2023-05-29

**Authors:** Javier de la Rubia, Rafael Alonso, María Esther Clavero, Elham Askari, Alfonso García, Cristina Antón, Margarita Fernández, Fernando Escalante, Ana García, Rafael Rios-Tamayo, Venancio Conesa, María Arancha Bermúdez, Beatriz Merchán, Alberto E. Velasco, María Jesús Blanchard, Antonia Sampol, Eukene Gainza, Prisma Montserrat Hernández, Adrián Alegre

**Affiliations:** 1 Hospital Universitario y Politécnico La Fe & Universidad Católica de Valencia, Centro de Investigación Biomédica en Red de Cáncer, CIBERONC CB16/12/00284, Instituto de Salud Carlos III, 28029 Madrid, Spain; 2 Department of Hematology, University Hospital La Fe and Universidad Católica de Valencia, Avda. Fernando Abril Martorell, 106, 46026 Valencia, Spain; 3 Hospital 12 de Octubre, 28041 Madrid, Spain; 4 Hospital Virgen de las Nieves, 18014 Granada, Spain; 5 Fundación Jiménez Díaz, 28040 Madrid, Spain; 6 Hospital Clínico, 47003 Valladolid, Spain; 7 Hospital Morales Meseguer, 30008 Murcia, Spain; 8 Hospital Reina Sofía, 14004 Córdoba, Spain; 9 Hospital de León, 24071 León, Spain; 10 Hospital Dr. Peset, 46017 Valencia, Spain; 11 Hospital Puerta de Hierro, 28222 Madrid, Spain; 12 Hospital General de Elche, 03203 Alicante, Spain; 13 Hospital Marqués de Valdecilla, 39008 Santander, Spain; 14 Hospital Universitario de Guadalajara, 19002 Guadalajara, Spain; 15 Hospital Rey Juan Carlos, Móstoles, 28933 Madrid, Spain; 16 Hospital Ramón y Cajal, 28034 Madrid, Spain; 17 Hospital Son Espases, 07020 Palma de Mallorca, Spain; 18 Hospital de Galdakao, 48960 Bilbao, Spain; 19 Hospital de S. Pedro, 26006 Logroño, Spain; 20 Spain for the Spanish Myeloma Group, Hospital Universitario La Princesa, 28006 Madrid, Spain

**Keywords:** belantamab mafodotin, multiple myeloma, relapse, refractory, treatment

## Abstract

**Simple Summary:**

Patients with multiple myeloma (MM) who become refractory to three or more lines of therapy (RRMM patients) have few valid therapeutic alternatives. Among them, drugs directed against the BCMA antigen expressed in plasma cells are very appealing. Belantamab-mafodotin (belamaf) is the first antibody-drug conjugate against BCMA ready for clinical use. In this paper, we report the Spanish experience of belamaf monotherapy in 156 patients with RRMM. The overall response rate was 41.8%, with 39.8% of patients achieving a partial response or better. Median progression-free survival was 3.61 months, but interestingly, it increased to 14.47 months in patients achieving a minimal response or better. Treatment was well tolerated, ocular events being the most reported toxicity (87.9%; grade ≥ 3, 33.7%), but only two patients discontinued treatment due to side effects. Overall, our results confirm the safety and efficacy of belamaf in this poor prognosis subset of patients.

**Abstract:**

Belantamab-mafodotin (belamaf) is a novel antibody-drug conjugate targeting B-cell maturation antigen that showed anti-myeloma activity in patients with relapsed and refractory multiple myeloma (RRMM). We performed an observational, retrospective, and multicenter study aimed to assess the efficacy and safety of single-agent belamaf in 156 Spanish patients with RRMM. The median number of prior therapy lines was 5 (range, 1–10), and 88% of patients were triple-class refractory. Median follow-up was 10.9 months (range, 1–28.6). The overall response rate was 41.8% (≥CR 13.5%, VGPR 9%, PR 17.3%, MR 2%). The median progression-free survival was 3.61 months (95% CI, 2.1–5.1) and 14.47 months (95% CI, 7.91–21.04) in patients achieving at least MR (*p* < 0.001). Median overall survival in the entire cohort and in patients with MR or better was 11.05 months (95% CI, 8.7–13.3) and 23.35 (NA-NA) months, respectively (*p* < 0.001). Corneal events (87.9%; grade ≥ 3, 33.7%) were the most commonly adverse events, while thrombocytopenia and infections occurred in 15.4% and 15% of patients, respectively. Two (1.3%) patients discontinued treatment permanently due to ocular toxicity. Belamaf showed a noticeably anti-myeloma activity in this real-life series of patients, particularly among those achieving MR or better. The safety profile was manageable and consistent with prior studies.

## 1. Introduction

Belantamab mafodotin (belamaf) is the first antibody-drug conjugate directed against B-cell maturation antigen (BCMA) available for use in patients with multiple myeloma (MM) [1,2]. Together with the chimeric antigen receptor T cells and bispecific antibodies forms, belamaf is one of the three pillars of immunotherapy used in the management of MM patients, particularly with advanced stages of the disease, i.e., those who are refractory to the three classes of conventional drugs administered in the management of MM: proteasome inhibitors, immunomodulatory drugs, and monoclonal antibodies. In the phase 2 randomized trial DREAMM-2, 97 patients receiving a median of six prior lines of therapy were allocated to the 2.5 mg/kg dose. The achieved ORR was 31%, and the median progression-free survival (PFS) was 2.9 months [3]. In a later update with a median follow-up of 13 months, the median estimated duration of response (DoR), overall survival (OS), and PFS were 11.0 months, 13.7 months, and 2.8 months, respectively [4].

Randomized phase 2 or phase 3 studies remain the ‘gold standard’ for obtaining regulatory approvals, based on their strong internal validity, prespecified and well-defined endpoints, and use of randomization, blinding, and control arms. However, these prospective studies have limitations in terms of external validity and generalizability and application in a real-world setting, including the fact that the feasibility of and adherence to these regimens may be limited due to varying patient-, treatment-, and disease-related factors. Furthermore, approximately 40% of real-world MM patients do not meet the criteria for randomized clinical trials on which approvals are based [5,6,7,8].

Therefore, to provide a more holistic definition of the effectiveness of a regimen in a real-world setting, treatment decisions must be tailored based on additional considerations beyond clinical trial efficacy. In this regard, since its availability, several groups have reported the results of belamaf monotherapy in different settings of real-world patients [9,10,11,12,13,14,15]. Consequently, we have retrospectively investigated the profile of and treatment patterns and outcomes in patients with RRMM managed with single-agent belamaf in a series of 156 consecutive patients included in the expanded access program or the compassionate use program available in Spain between November 2019 and June 2021.

## 2. Methods and Patients

This is an observational, multi-center, retrospective study performed on patients with RRMM from 84 Spanish hospitals who received treatment with single-agent belamaf under GSK expanded access or compassionate use between November 2019 and June 2021. Criteria for the inclusion of patients in the program were as follows: Patients aged 18 years or older provided written informed consent, diagnosis of RRMM according to International Myeloma Working Group criteria [16], failure of ≥4 prior therapies, refractoriness to an anti-CD38 antibody (if available) alone or in combination, to an immunomodulatory drug and to a proteasome inhibitor, and had disease progression on last therapy.

Every patient received belamaf at a dose of 2.5 mg/kg every 3 weeks until disease progression or unacceptable toxicity. In case of adverse events ≥ grade 2, the dose was delayed until recovery to grade 1 or less. No premedication was administered. The primary end point of this study was the overall response rate (ORR) defined as the best overall confirmed response of minimal response (MR), partial response (PR), very good partial response (VGPR), complete response (CR), or stringent complete response (sCR) as reported by the investigator. Secondary objectives were to estimate the time to response (TTR), time to next treatment (TTNT), progression-free survival (PFS), overall survival (OS), duration of response (DoR), and PFS2. From a safety point of view, data on non-ocular treatment-emergent adverse events (TEAEs) were collected and assessed using CTCAE v5.0. Furthermore, the type, incidence, severity, and rate of recovery of ophthalmologic AEs were evaluated by ophthalmologic evaluation by an ophthalmologist at the beginning of each cycle, at least for the first four cycles.

### Statistical Analysis

Categorical variables were compared by the chi-square test or by the Fisher exact test, and continuous variables were tested by the T test and checked by the Mann-Whitney U test. When calculating the proportion of patients achieving an overall response, patients with unknown or missing response data were treated as non-responders. PFS was calculated for all patients from the day of the first dose of belamaf to relapse or death from any cause, and OS was defined as the time interval from the day of starting belamaf until death from any cause, with survivors censored at the last follow-up. DoR, PFS, OS, and PFS2 were plotted according to the Kaplan-Meier method, with comparisons made by the log-rank test. Differences were considered statistically significant when *p*-values were <0.05 in a two-tailed test. All analyses were performed by the Statistical Package for the Social Sciences (SPSS) v.24 and R version 2.13.2 (R Development Core Team, 2011, R Foundation for Statistical Computing, Vienna, Austria).

## 3. Results

At the time of the study, a total of 166 patients from 84 hospitals received belamaf as part of the compassionate use program or the expanded access program in Spain. Ten patients (6%) did not enter in the study due to investigators’ decision, based on logistic issues not otherwise specified, leaving 156 patients to be finally included in this analysis.

The characteristics of the patients at baseline are shown in Table 1. The median age at study entry was 72.5 years (range, 40–89), and 84 (53.8%) were female. The high-risk cytogenetics profile (defined as the presence of any of the following cytogenetics: t(4;14), t(14;16), t(14;20), 17p13del, or 1q21+) is shown in Table 1. Nineteen patients (12.2%) had a creatinine clearance < 30 mL/min, and extramedullary disease (EMD), defined as the presence of one or more extramedullary soft tissue lesions, was present in forty-nine patients (31.4%). Patients had received a median of five (interquartile range [IQR], 4–6) lines of treatment before administration of belamaf, and the median time between diagnosis and the first dose of belamaf therapy was 6.4 years (IQR, 3.7–8.3). A total of 129 out of 149 patients with data available (82.7%) had received at least two immunomodulatory agents, at least two proteasome inhibitors, and at least one anti-CD38 antibody (penta-drug exposure). No patient received prior anti-BCMA therapy. Before study entry, 125 patients (80.1%) had triple-refractory disease, and 54 (34.6%) were penta-refractory. Finally, 101 patients (64.7%) had previously undergone autologous stem cell transplantation. Other characteristics of the patients are presented in Table 1.

### 3.1. Efficacy

**Figure 1:**
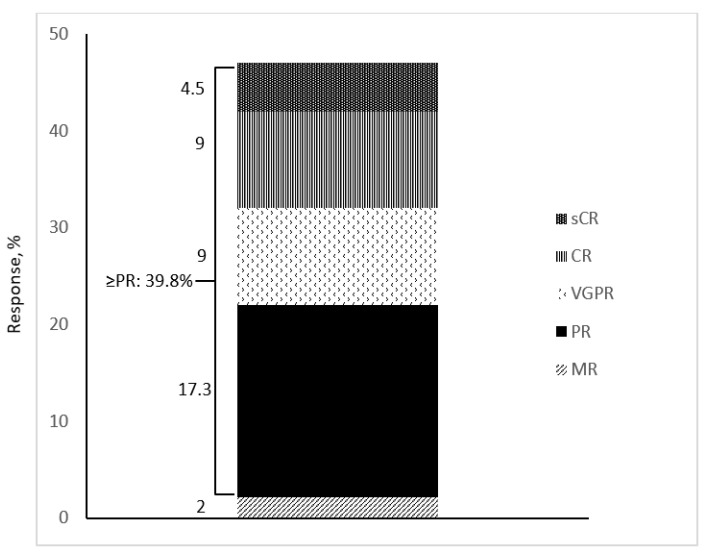
Overall response rate.

The ORR was 41.8% (65 patients), while 62 patients (39.8%) achieved PR or better (Figure 1).

A VGPR or better was achieved by 35 (22.5%) patients, which included sCR or CR in 21 patients (13.5%). The median TTR in patients achieving ≥MR was 1 month (range, 1–2.5), and the median time to the best response in responders was 4.7 (range, 1–23.4) months. The overall proportions of patients achieving ≥MR according to different characteristics are shown in Table 2. At the data cutoff date (31 July 2022), 26 (16.7%) patients continued to be on treatment.

### 3.2. PFS, DoR, OS, and TTNT

**Figure 2:**
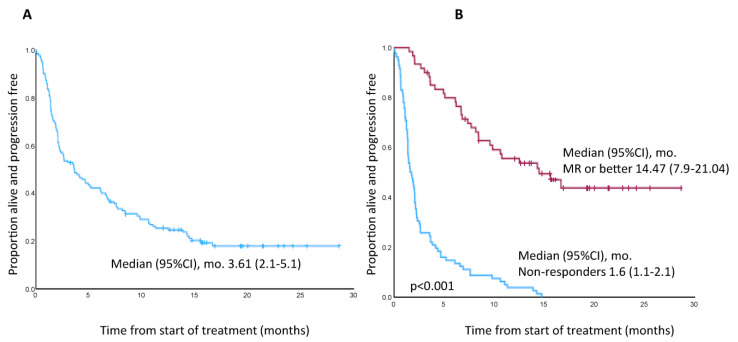
(**A**) Progression-free survival in the overall series. (**B**) Progression-free survival in patients achieving minimal response or better versus non-responders.

The median follow-up was 10.9 months (range, 1–28.6). The median duration of PFS in the overall series was 3.61 months (95% Confidence Interval [CI], 2.1–5.1) (Figure 2A). 

Median PFS in non-responding patients and in patients achieving at least MR was 1.6 (95% CI, 1.1–2.1) and 14.47 months (95% CI, 7.91–21.04), respectively (*p* < 0.001) (Figure 2B). 

PFS by response category is illustrated in Supplementary Figure S1A. Among triple-refractory patients, median PFS was 2.66 months (95% CI, 1.5–3.8) vs. 6.9 months in non-triple refractory patients (95% CI, 1.1–12.6; *p* = 0.043) (Supplementary Figure S1A). In responding patients (at least MR), median DoR was 13.9 months (95% CI, 8.3–19.4) (Supplementary Figure S2A).

**Figure 3:**
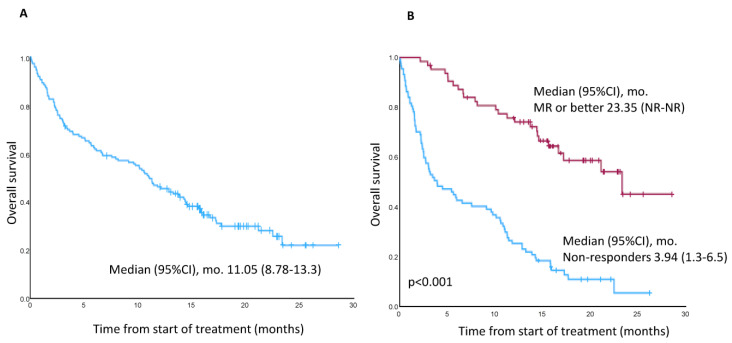
(**A**) Overall survival of total cohort of patients. (**B**) Overall survival in patients achieving minimal response or better versus non-responders.

The median duration of OS in the all-treated population was 11.05 months (95% CI, 8.78–13.3) (Figure 3A), and the OS in patients achieving at least MR vs. non-responders is shown in Figure 3B.

OS by response category is shown in Supplementary Figure S1B. There was a trend towards a longer OS among patients who did not have EMD at study entry versus those who did (13.2 months [95% CI, 10.3–16.1] vs. 4.7 months [95% CI, 0–10.3]; *p* = 0.089), while OS in patients with a CrCl <30 mL/min and >30 mL/min were 5.1 (95% CI, 0–21.1) and 11.05 (95% CI, 8.7–13.3) months, respectively. Finally, OS was similar in triple-refractory (10.6 months; 95% CI, 7.4–13.8) vs. non-triple-refractory (11.44 months; CI 95% CI, 8.2–14.69) patients. Overall, 105 (67.3%) patients have died. Causes of death were disease under study (n = 95) and unknown (n = 10).

A total of 64 of the 153 patients with available data received at least one further anti-myeloma therapy after relapse from belamaf. In every case, the regimens administered included drugs already administered prior to belamaf (Supplementary Table S1). The median TTNT in this group of patients was 4.0 months (range, 1.1–15.3), and median PFS2 was 8.15 months (95% CI, 4.3–11.9) (Supplementary Figure S2B).

### 3.3. Safety

#### 3.3.1. Ocular Toxicity

Overall, 56 (36%) patients had some type of ocular disease before receiving treatment with belamaf. Information on ocular side effects was available in 83 out of the 154 patients (53.2%). Corneal events, reported in 73 patients (87.9%), were the most observed TEAE, G1-2 in 45 patients and ≥G3 in 28. Improvement to ≤G1 was observed in 56 out of the 70 patients with available data, while in the remaining patients some degree of corneal damage was still present at the end of the study. In addition, corneal events recurred in 23 patients after re-exposure to belamaf, with 5 patients presenting more than three recurrences, 10 patients suffering two recurrences, and 8 patients one recurrence.

Fifty-two (33.3%) patients reported at least one ocular symptom during therapy with belamaf, a reduction of visual acuity being the most observed (50 patients). According to the severity, it was ≥G3 in 7 patients, G2 in 17 patients, and G1 in 1 patient (no information was available in the remaining cases). Visual acuity was fully recovered in all but 7 patients. Other reported symptoms are shown in Table 3. Finally, only 2 (1.3%) out of the 156 patients included in the study discontinued treatment permanently due to ocular toxicity.

#### 3.3.2. Non-Ocular Toxicity

A total of 66 patients (42.3%) developed 129 non-ocular TEAEs, and 35 patients were ≥G3. The most common hematologic adverse events were thrombocytopenia (in 24 patients [15.5%]), neutropenia (in 7 [4.4%]), and anemia (in 6 [3.8%]). Infections occurred in 14 patients (9%), and 9 of them (5.6%) had ≥3 infectious episodes. The remaining observed AEs were each reported in <5% of patients (Table 3). TEAEs resulting in treatment discontinuation were observed in six patients. The causes for discontinuation were keratopathy (two cases), liver toxicity, thrombocytopenia, tumoral lysis syndrome, and cardiac toxicity (one each).

## 4. Discussion

Our study includes the largest series, so far reported, of RRMM patients receiving single-agent belamaf at the standard dose of 2.5 mg/kg treated outside a clinical trial. It represents 94% of the patients treated in Spain, reflecting the experience of the use of this drug in real-world practice in our country.

The ORR, defined as at least MR, was 41.7% consistent with those reported by other groups including treatment with belamaf monotherapy in a real-world population of patients (see Table 4) [9,10,11,12,13,14,15]. Likewise, the rate of PR or better observed in our series (39.8%) is also like the results observed by most of the other groups, which range from 46% to 32.7% [9,10,11,12,13,14,15]. Interestingly, the ALFA study is the only one that reports no CR, probably because bone-marrow examinations were not routinely performed [12]. These findings, however, contrast with the ORR of 31% and 33% reported in the 2.5 mg/kg cohort of the pivotal DREAMM-2 study and in the series by Vaxman et al., respectively [3,9]. Although these results might initially seem inferior, the patient groups recruited to DREAMM-2 and in the study of the Mayo Clinic are somewhat different from those recruited in other real-world series, having all been exposed and refractory or intolerant to daratumumab, whereas the proportion of patients refractory to an anti-CD38 antibody was 82.7% in our study and 80.1% in the study from Shragai et al. [11]. Finally, there are no data available on the status of anti-CD38 exposure and/or refractoriness in the remaining series.

Our series is the only one that analyzes the response of patients with severe renal impairment, and the ORR in our analysis was similar in patients regardless of the creatinine clearance. However, no specific analysis of patients with renal impairment is available in the remaining real-world series, and only five patients with severe renal impairment (creatinine clearance between <30 and ≥15 mL/min) were included in the DREAMM-2 trial [3]. Therefore, no definitive conclusions can be provided about the efficacy and safety of belamaf in this population of patients. Finally, our study included 49 (31.4%) patients with plasmacytomas. Contrary to the results reposted by Lonial et al. [2], we observed an ORR in these patients similar to that of patients without EMD, but with a shorter OS (4.7 months vs. 13.2 months; *p* = 0.089), suggesting a limited efficacy of monotherapy with belamaf in this subset of patients. The ALFA study also included 15 patients with EMD, and no response was observed in any of them [12].

As with the ORR, the PFS reported with treatment with single-agent belantamab in the different series was very similar, ranging from 2.4 months in the ALPHA study to 4.9 months in the study by Atieh et al., while in our study the median PFS was 3.6 months [9,10,11,12,13,14,15] (Table 4). Interestingly, however, there also seems to be a slightly reduced duration of PFS in some of the series with 100% of patients refractory to anti-CD38 antibodies [3,10], but not in others [11]. Finally, contradictory results have been reported regarding the role of prior BCMA therapy in patients treated with belamaf. Vaxman et al. reported a PFS of only 2 months in their series of 36 patients, including 7 with previous CAR-T cell therapy [9]. On the other hand, in the study by Hultcrantz et al. that included 17 patients exposed to BCMA therapy, the response rate and the duration of PFS after belamaf were similar to other series that did not involve BCMA-treated patients [15]. However, the low number of patients precludes drawing definitive conclusions, and this finding needs to be confirmed in larger series of patients.

As expected, those patients who achieved at least MR in our study had a longer median PFS than those who did not (14.4 months vs. 1.6; *p* < 0.001). This finding has been reported in other series, with an estimated median PFS in patients who achieved a VGPR of 14 months and 20.6 months in the DREAMM-2 and ALFA studies, respectively [3,12], while in the series by Shragai et al. patients achieving at least PR also had a significant benefit in terms of PFS versus those who did not [11].

The toxicity profile of belamaf is very characteristic, and it has been reproduced in every study that has included this drug. Corneal events have been related to monomethyl auristatin F, the cytotoxic payload of belamaf [18], and this is the most common ocular toxicity reported after treatment with single-agent belamaf [3,9,10,11,12,13,14,15]. In our study, data on corneal events were available in 83 patients, and 73 of them (87.9%) developed this complication. These results are slightly higher than those reported by the DREAMM-2 trial and by other real-world series [3,11,13,14,19]. The rate of corneal events ≥G3 in our series was 33.7%, ranging from 8.2% in the MAYO study to 56% in the series by Atieh et al., though this last report only included 28 patients [9,12]. Only two patients in our analysis required permanent discontinuation due to ocular toxicity, and discontinuation rates below 10% have been reported by most real-world data series [10,11,13,14], with only one series including 38 patients depicting a discontinuation rate secondary to ocular toxicity of 14% [20]. These discrepancies could be due to several factors, such as the degree of experience with this drug, the capability to access periodic ophthalmologic evaluations, and the existence of different baseline ocular abnormalities in the patients, among others. However, undoubtedly, belantamab-associated ocular adverse events are the most characteristic toxicity of this drug, influencing adherence to treatment and compromising efficacy due to frequent interruptions of the drug. Therefore, aiming to minimize the incidence and severity of these complications, some authors have suggested that doses of belamaf and/or longer intervals between doses may result in a reduction in ocular AEs and an improvement of the anti-MM efficacy of the treatment [21,22]. In addition, results of the DREAMM-14 trial (NCT05064358), which analyzes alternative doses and schedules of belamaf, will also be of help in identifying measures aimed at reducing the incidence and severity of this complication [23].

The observed incidences of other common non-ocular TEAEs of interest were also similar to prior series, including thrombocytopenia and infections [3,11]. Interestingly, however, we observed two cases of severe liver toxicity and one case of tumor lysis syndrome leading to permanent drug discontinuation, a finding also reported by other groups [11]. Finally, no new safety concerns were observed in our study.

Recently, the French cooperative IFM group has prepublished their experience with single-agent belamaf in a series of 106 patients with RRMM and at least three lines of prior therapy. Overall, 106 patients were included in the study, the median PFS and OS were 3.5 months (95% CI, 1.9–4.7) and 9.3 months (95% CI 5.9,15.3), respectively, and ORR was 38.1% [17]. Ophthalmic adverse events were also the most observed toxicity (48%), being keratopathy in 37.5% of cases (Table 4). Finally, the results of the phase 3 DREAMM-3 trial comparing single-agent belamaf vs. pomalidomide plus dexamethasone in patients with RRMM have been recently reported. After a median follow-up of 11.5 months, the median PFS with belamaf was 11.2 months vs. 7 months with pomalidomide and dexamethasone (HR, 1.03), not meeting the primary endpoint of PFS superiority [24]. However, it is important to note that the response achieved by the patients treated with belamaf was more durable and deeper, with no new safety signals identified. Furthermore, the patient population of this study does not represent the unmet need of the triple-class refractory patients that were included in our study, due to a 40% enrolment of participants without prior anti-CD38 treatment.

Bispecific antibodies are other off-the-shelf anti-BCMA therapies for patients with RRMM [25,26,27]. Though no trials are comparing these T-cell redirecting antibodies against belamaf, the available data suggest that bispecific antibodies show higher anti-myeloma activity than belamaf [28,29,30]. However, the choice between both alternatives may be affected by different factors. For example, the safety profile, particularly the higher incidence of infections associated with the prolonged administration of these drugs, is a matter of concern and could affect the final decision regarding which type of therapy to administer to different subgroups of patients.

The strength of our series is that it is one of the largest ones to be focused on patients with RRMM treated outside clinical trials, including special populations, such as those with renal impairment or extramedullary disease. Therefore, it reflects the efficacy of monotherapy with belamaf in the real world. However, it also has several limitations. This study was retrospectively designed. Therefore, we may have missed some information, particularly related to side effects. For example, the tools used to evaluate ocular side effects among the different participating centers were not uniform, and we did not routinely collect relevant information on the management of ocular toxicity (i.e., number of dose reductions or dose delays of belamab). Consequently, the different approaches to treating this complication between centers could impact the results.

## 5. Conclusions

In conclusion, our study confirms the activity of single-agent belamaf in a real-world setting, with patients achieving at least MR showing prolonged PFS. As previously reported, corneal events, involving most of the patients, were the most common non-hematological toxicities observed. Though these complications led to permanent drug discontinuation in a minority of patients, in our series more than one third of patients developed reduced visual acuity or blurry vision, and ocular toxicities remain a major challenge in treatment with belamaf. Finally, further studies on earlier lines of therapy with newer regimens, including belamaf, will contribute to confirming the role of this drug in the current MM scenario.

## Figures and Tables

**Table 1:** Baseline patient characteristics and demographics.

Characteristics		n = 156
Age at diagnosis, median (IQR)		70 (64–76.8)
Sex (female), no. (%)		84 (53.8)
Time from diagnosis, years, median (IQR)		6.4 (3.7–8.3)
Creatinine clearance (mL/min), no./total no. (%)		
	≥30/<30	130 (83.3)/19 (12.2)
Type of MM, no. (%)		
	IgG/Non-IgG	84 (53.9)/72 (46.1)
ECOG performance status score, no./total no. (%)		
	0/≥1	34 (21.8)/104 (66.7)
ISS at diagnosis, no./total no. (%)		
	I/II/III	45 (28.8)/49 (31.4)/52 (33.3)
R-ISS at diagnosis, no./total no. (%)		
	I/II/III	33 (21.2)/53 (34.0)/32 (20.5)
EMD, no./total no. (%)		49 (31.4)
High-risk cytogenetics, no./total no. (%)		
	del(17p)	17/73 (23.3)
	t(4;14)	15/69 (21.7)
	t(14;20)	1/51 (2)
	1q21+	28/60 (46.7)
Prior treatments, median (IQR)		5 (4–6)
Refractory status, no. (%)		
Refractory to ≥1 line of treatment, no. (%)		146 (93.6%)
	To IMiDs	133 (85.3)
	To PI	131 (84)
	To anti-CD38 MoAbs	129 (82.7)
	Triple-refractory	125 (80.1)
	Penta-refractory	54 (34.6)
Refractory to last line of therapy		123 (83.1)
Previous HSCT, no. (%)		101 (64.7) ^a^

IQR: interquartile range; EMD: extramedullary disease; IMiDs: immunomodulatory drugs; PI: proteosome inhibitor; MoAbs: monoclonal antibodies; HSCT, hematopoietic stem cell transplantation. ^a^ All autologous.

**Table 2:** Response rates, progression-free survival, and overall survival by subgroups.

Subgroup, No.		ORR, %	PFS (95% CI), mo.	OS (95% CI), mo.
Refractoriness				
	Triple-refractory, 125	60.8	2.6 (1.5–3.8)	10.62 (7.4–13.8)
	Non-triple refractory, 23	69.6	6.9 (1.1–12.6)	11.4 (8.2–14.7)
	Penta-refractory, 54	62.8	2.2 (0.5–3.8)	9.77 (0.6–18.8)
	Non-penta refractory, 94	61.1	4.9 (2.3–7.4)	11.05 (9.4–12.6)
Age				
	≤70 years, 71	62	2.6 (0.7–4.5)	10.01 (5.4–14.7)
	>70 years, 83	62.7	3.6 (1.9–5.3)	12.07 (9.1–14.9)
CrCl				
	<30 mL/min, 17	41.2	2.07 (1.7–2.3)	5.09 (0–21.1)
	≥30 mL/min, 130	43.8	4.1 (2.7–5.5)	11.05 (8.7–13.3)
EMD				
	No, 106	44.3	4.67 (2.4–6.8)	13.2 (10.3–16.1)
	Yes, 49	37.5	2.1 (1.3–2.8)	4.7 (0–10.3)

CrCl: Creatinine clearance; EMD: Extramedullary disease.

**Table 3:** Most frequently observed treatment-emergent adverse events.

		All Grades, No. (%)	≥Grade 3, No. (%)
Hematologic			
	Thrombocytopenia	24 (15.4)	17 (10.9)
	Neutropenia	7 (3.8)	5 (3.1)
	Anemia	6 (3.9)	2 (1.3)
Non-hematologic			
	Infections	25 (15)	10 (5.6)
	Gastrointestinal	9 (5.8)	2 (1.2)
	Hepatobiliary	8 (5.1)	3 (1.8)
	Neurological	6 (3.9)	2 (1.2)
	Fatigue	5 (3.2)	0
	Cardiac & Vascular	5 (3.2)	1 (0.6)
	Respiratory	4 (2.4)	2 (1.2)
	Metabolic	3 (1.9)	0
	Renal	3 (1.9)	2 (1.2)
	Other	10 (6.2)	1 (0.6)
Ocular ^a^			
	Corneal events	73 (87.9)	28 (33.7)
	Reduced visual acuity	50 (60.2)	7 (8.4)
	Blurry vision	31 (37.3)	0
	Dry eye	27 (32.5)	0
	Foreign body sensation	16 (19.2)	0
	Ocular discomfort	15 (18.1)	0
	Photophobia	10 (12)	0
	Other	7 (8.4)	0

^a^ Data available for 154 patients. Other: Cataracts (2), corneal deposits (2), blepharitis (1), diplopia (1).

**Table 4:** Studies of single-agent belantamab mafodotin in patients with relapsed/refractory multiple myeloma.

Author, Year	No. of Patients	Age, Years (Range)	No. of Lines (Range)	Triple-ref, %	Penta-refr, %	ORR, % ^a^	≥PR, %	PFS (95% CI), mo.	DoR (95% CI), mo.	OS (95% CI), mo.
Lonial, 2020 [3]	97	65 (60–70)	7 (3–21)	100	NA	35	31 ^b^	2.8 (1.6–3.6)	11 (4.2–NR)	13.7 (9.9–NR)
Vaxman, 2021 [9]	36	61 (37–83)	8 (7–11)	100	100	NA	33	2 (NA)	NA	6.5 (NA)
Iula, 2022 [10]	28	67.5 (51–83)	6 (3–14)	100	NA	NA	40	3 (0–23)^c^	NR (2–23)	8 (0–23)
Shragai, 2022 [11]	106	69.4 (36.3–80)	6 (2–11)	72.6	32	NA	45.5	4.7 (3.5–5.9)	8.1 (5.7–10.5)	14.5 (9.5–19.6)
Atieh, 2021 12]	28	67 (24–85)	5 (3–15)	100	54	46	46	4.9 (NA)	NA	7.4 (NA)
Rousell, 2022 [13]	184	70.3 (63.3–75.9)	5 (2->5)	NA	NA	36.4	32.7	2.4 (1.9–3.2)	NA	8.8 (6.3–11.6)
Offidani, 2022 [14]	67	66 (42–82)	5 (2–10)	NA	NA	37	31	3.7 (NA)	13.8 (NA)	12.9 (NA)
Hultcrantz, 2022 [15]	90	66 (37–88)	6 (2–14)	NA	NA	63 ^d^	42	4 (NA)	13.1 (NA)	20.5 (NA)
Talbot, 2023 [17]	106	66 (37–82)	5 (3–12)	56.7	11.3	38.1	NA	3.5 (1.9–4.7)	9 (4.65–10.4) ^c^	9.3 (5.9.15.3)
This series, 2023	156	72.5 (40–89)	5 (2–10)	80.1	34.6	41.8	39.8	3.6 (2.1–5.1)	13.9 (8.3–19.4)	11.05 (8.7–13.3)

ORR, overall response rate; PR, partial response; PFS: progression-free survival; DoR: duration of response; OS: overall survival; mo, months; NA: not available; NR: not reached. ^a^ ≥MR; ^b^ ≥VGPR; ^c^ Range; ^d^ ≥Stable disease.

## Data Availability

The data that support the findings of this study are available from the Spanish cooperative group PETHEMA, but restrictions apply to the availability of these data, which were used under license for the current study and are therefore not publicly available. Data are however available from the authors upon reasonable request and with permission of PETHEMA.

## Supplementary Materials

The following supporting information can be downloaded at: https://www.mdpi.com/article/10.3390/cancers15112964/s1, Table S1: Treatments administered after therapy with belantamab mafodotin; Figure S1: (A) Progression-free survival by response category. (B) Overall survival by response category; Figure S2: (A) Duration of response. (B) Progression-free survival 2.
