# Supplementary material for: Belantamab Mafodotin in Patients with Relapsed/Refractory Multiple Myeloma: Results of the Compassionate Use or the Expanded Access Program in Spain
Source: Cancers (Basel). 2023 May 29;15(11):2964. doi: 10.3390/cancers15112964 (PMC10251953; doi:10.3390/cancers15112964)

Supplementary Table S1. Treatments administered after therapy with belantamab mafodotin

Treatment	No. of patients	%
PI-based	18	28.1
IMiD-based	10	15.6
Anti-CD38 MoAb-based	2	3.1
Combination of PI + IMiD	7	11
Combination of PI + IMiD + Anti-CD38 MoAb	1	1.6
Other regimens	26	40.6

Note: Treatments and values thereof are based on the treatment patient received at first therapy after belantamab. Frequencies are based on N=64 patients. Abbreviations: PI, proteosome inhibitor; IMiD, immunomodulatory drug; MoAb, monoclonal antibody.

Supplementary Figure S1. (A) Progression-free survival by response category. (B) Overall survival by response category.

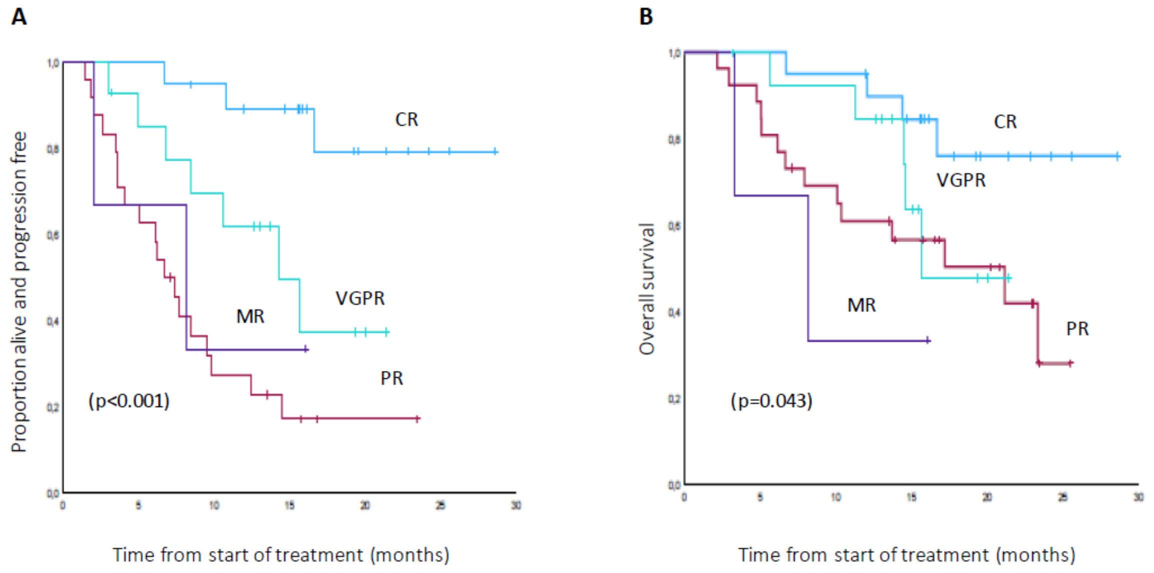

Supplementary Figure S2. (A) Duration of response. (B) Progression-free survival 2.

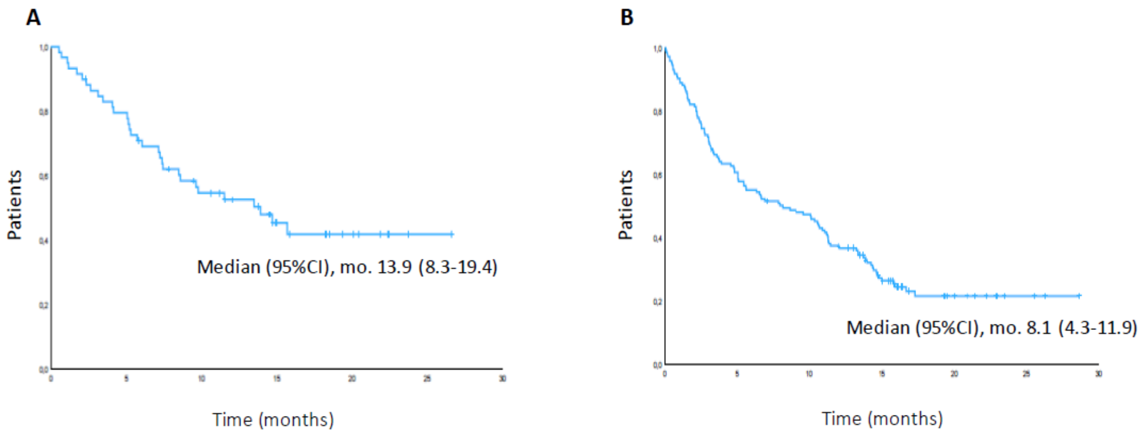

Supplement: Supplementary file 1 [file cancers-15-02964-s001.zip › cancers-2384734-supplementary.pdf]
